# Case Report: Bocavirus Infection Radiologically Resembling a Congestive Heart Failure in a Patient with Metastatic Castration-Resistant Prostate Cancer

**DOI:** 10.12688/f1000research.109221.2

**Published:** 2022-03-29

**Authors:** Javier David Benitez Fuentes, Alicia de Luna Aguilar, Paloma Flores Navarro, Alfonso Lopez de Sa Lorenzo, Carmen Toledano Rojas, Berta Laguna Fonseca, Richa Shah, Clara Frick, Alejandro Francisco Jimenez Ortega, Tania Rocha Iñigo, Natalia Vidal Cassinello

**Affiliations:** 1 Departamento de Oncología Médica, San Carlos University Hospital, IdISSC, Madrid, Madrid, 28040, Spain; 2 Johns Hopkins Bloomberg School of Public Health, Baltimore, MD, 21205, USA; 3 Departamento de Microbiología, San Carlos University Hospital, IdISSC, Madrid, Madrid, 28040, Spain; 4 Cancer Surveillance Branch, International Agency for Research on Cancer, Lyon, 69008, France; 5 Public Health, Ludwig Maximilian University of Munich, Munich, Germany; 6 Farmacología Clínica, San Carlos University Hospital, IdISSC, Madrid, Madrid, 28040, Spain; 7 San Miguel Radiology Center, Badajoz, 60612, Spain

**Keywords:** Case Report, Bocavirus, Respiratory Tract Infection, Prostate Cancer, Immunocompromised Host, Emerging Communicable diseases

## Abstract

**Background: **Human bocavirus (HBoV) is a viral pathogen from the genus *Bocaparvovirus* (family *Parvoviridae*, subfamily *Parvovirinae*) discovered in 2005. Most of available literature is about HBoV in children and adults with hematological malignancies and in otherwise healthy children with respiratory infections. Information regarding infection in the adult population with solid tumors is scarce.

**Case Report: **We report the case of a 51-year-old male with metastatic castration resistant prostate cancer undergoing chemotherapy treatment who presented with fever, dyspnea, dry cough, and pleuritic pain. Imaging techniques showed signs of congestive heart failure. Symptoms, laboratory tests and echocardiography revealed a more probable infectious etiology. Antibiotic therapy was started. A polymerase chain reaction (PCR) test of nasopharyngeal exudate for respiratory viruses was positive for HBoV. The rest of the microbiological tests were negative. Bronchoalveolar lavage (BAL) was performed. Bacterial culture of BAL was negative while respiratory virus PCR confirmed positivity for HBoV. Antibiotic therapy was discontinued. The patient gradually recovered.

**Conclusions: **Emerging infectious diseases are a notorious threat for immunocompromised populations such as solid tumor patients. This case is unique because to our knowledge this is the first case report article of HBoV in a solid tumor patient and because imaging techniques exhibited signs of congestive heart failure that did not correlate with the rest of the tests. It shows that unusual pathogens should be considered when managing serious clinical complications with uncommon presentations in cancer patients. Notable diagnostic efforts should be made to reach a diagnosis in these cases.

## Introduction

Human bocavirus (HBoV) is a single-stranded DNA viral pathogen belonging to the genus *Bocaparvovirus* (family *Parvoviridae*, subfamily *Parvovirinae*) which comprise four genotypes (HBoV1-4). ^1^ It was discovered in 2005 by Tobias Allander and coworkers at the Karolinska University Hospital, Stockholm, Sweden in nasopharyngeal aspirates from children with respiratory tract infection (RTI). ^2^ It can be found in respiratory secretions in high quantities during the acute phase and it can persist at low viral loads for months. ^3^ Besides respiratory samples, HBoV has been detected in feces, urine, saliva, blood, tonsils, and cerebrospinal fluid. ^3^ Since its discovery this pathogen has gained recognition as a virus with a wide global distribution. It has an estimated global prevalence of about 6%, depending on the region being studied its prevalence ranges from 1 to 56% of respiratory samples and from 1.3 to 63% of stool samples. ^3^

It is most likely transmitted by air and commonly associated with coinfection with other viruses making it difficult to assert if the main pathogen causing the symptoms is HBoV. ^4^ There is evidence that HBoV1 is associated with respiratory disease, especially in children. ^5^ HBoV1 has also been associated with long periods of persistence in the mucosa of the respiratory tract which might play a role in its frequency of co-infections with other well recognized respiratory pathogens. This concurrent detection of other viral respiratory pathogens is high, some studies show a concurrent detection rate of other viral respiratory pathogens in more than 50% of respiratory specimens. ^4^^,^^5^

Most of the published focus articles on HBoV infection in the pediatric population and case reports are usually about immunocompromised pediatric patients, especially pediatric hematopoietic cell transplant recipients, and hematologic malignancy pediatric patients. ^5^^–^^8^ Some studies have shown that HBoV is an uncommon pathogen in adult patients with severe pneumonia. ^9^ In many cases described, in the adult population, in literature HBoV is associated with increased mortality. ^9^ HBoV is closely associated with an immunocompromised state and severe comorbidities such as structural lung disease and hematologic malignancy. ^9^ Most original articles, case reports, and case series published in the immunocompromised adult population show hematopoietic cell transplant recipients and hematologic malignancies as comorbidities with few exceptions. There are few articles regarding the adult population with solid tumors. We found only one study describing the prevalence of HBoV in the adult population with solid tumors from Li *et al* ^10^ done in Wuhan (China) and published in 2011. This study revealed a prevalence percentage of HBoV infection in adult solid tumor patients of 39.74%. ^10^ In another study from Lee *et al* ^11^ done in Korea and published in 2019, a total of 185 adult subjects that were diagnosed with HBoV infection between January 2010 and December 2017 were enrolled into the study. Of these 185, 28 (15.13%) had solid tumors. ^11^ Their clinical characteristics and risk factors for pneumonia were retrospectively evaluated.

In this case report, we examine the case of a 59-year-old patient with a history of metastatic castration-resistant prostate cancer suffering from HBoV infection. To our knowledge, this is the first case report article of a solid tumor patient infected with HBoV.

## Case report

We report the case of a 59-year-old Caucasian male barbershop owner, former smoker, diagnosed with achalasia in January 2019 treated with Heller myotomy and Toupet fundoplication on the 6th of May 2021. In November 2019 he complained of bone pain at different anatomic locations and was diagnosed with metastatic prostate cancer and bone only disease.

The patient was started on androgen deprivation therapy, immunotherapy with ipilimumab and nivolumab as well as chemotherapy with docetaxel in December 2019 as part of a clinical trial ( NCT03879122). He initially received two cycles of intravenous ipilimumab 3 milligrams/kilogram (mg/kg) with intravenous nivolumab 3 mg/kg once a day on day one every three weeks for 6 weeks. Ipilimumab was discontinued due to grade three diarrhea, which was treated with a course of high dose oral steroids (equivalent to 2 mg/kg of prednisone) for two weeks until complete recovery. He then received four cycles of intravenous docetaxel 75 milligrams/square meter of body surface area (mg/m ^2^) with intravenous nivolumab (3 milligrams/kilogram) once a day on day one every three weeks for twelve weeks, followed by maintenance with intravenous nivolumab at the same dose and schedule as part of the study protocol. The treatment continued unchanged until November 2020 when it was stopped due to bone and serologic progression. After November 2020 the patient was started on abiraterone (1000 mg once a day) plus 5 milligrams of prednisone twice a day and received radiotherapy over the bone metastases in progression. In May 2021 treatment was changed due to serologic and bone progression to intravenous cabazitaxel (20 mg/m ^2^) once a day every three weeks plus prednisone 5mg twice a day every day. Dexamethasone 4 mg once a day everyday was added at the beginning of June 2021 due to bone pain.

**Figure 1.**
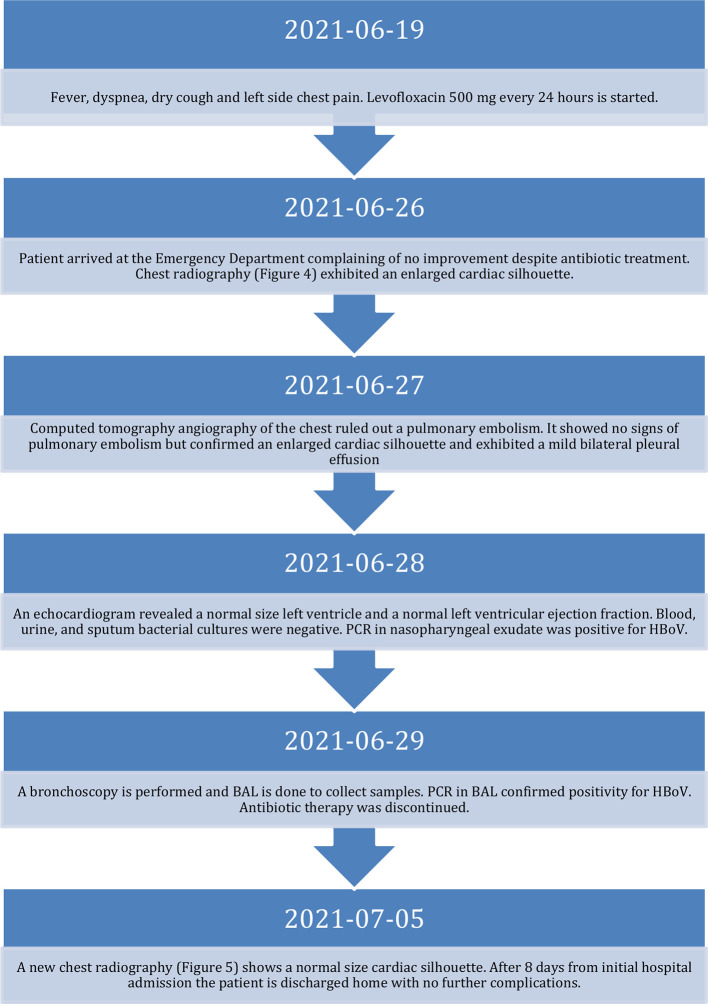
Timeline of events. PCR, polymerase chain reaction; BAL, bronchoalveolar lavage; HBoV, human bocavirus.

On the 26th of June 2021, the patient arrived at the emergency department complaining of fever at home, dyspnea, dry cough, and left side pleuritic chest pain for the last seven days. The sequence of events is detailed in the timeline ( Figure 1). Oral levofloxacin (500 mg) every 24 hours was started one week before the arrival of the patient to the emergency department with no improvement. His blood pressure was 90/50 mmHg (hypotension defined by 90/60 mmHg or below), temperature was 36.8°C (normal temperature level is between 36.1°C and 37.2°C), heart rate was 95 bpm (normal heart rate for adults 60 to 100 beats per minute), respiratory rate was 24 bpm (normal respiratory rate for adults 12 to 16 breaths per minute), peripheral oxygen saturation was 88% (normal level equal or more than 95%) with no supplemental oxygen.

Physical exam was pertinent with generalized decreased breath sounds with no signs of peripheral edema.

**Figure 2.**
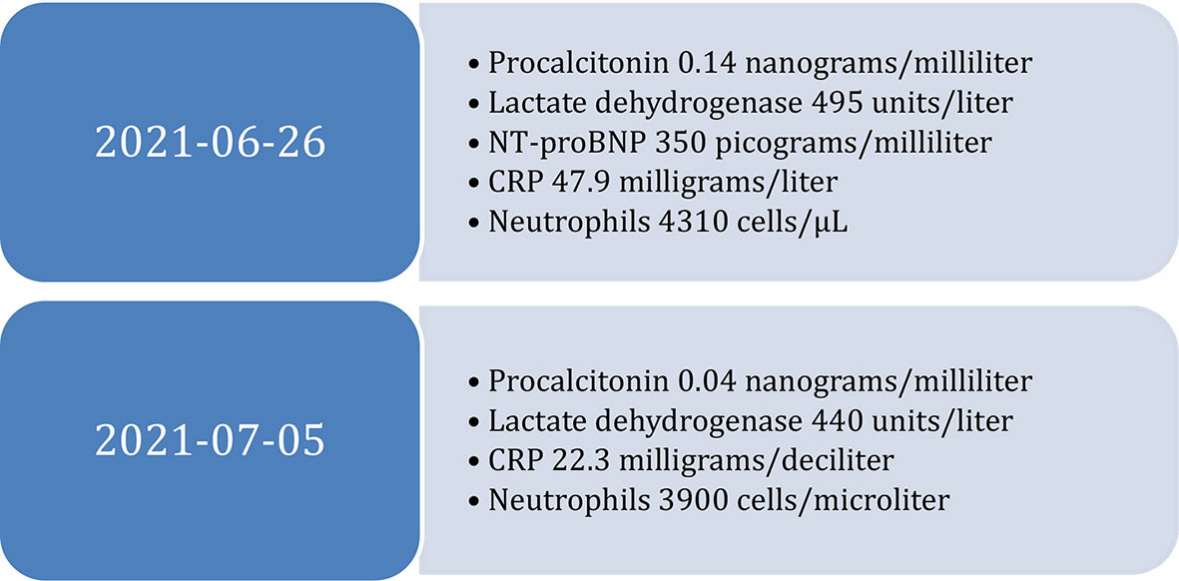
Diagnostic tests at admission and discharge. N-terminal pro-brain natriuretic peptide (NT-proBNP), C-Reactive Protein (CRP).

Laboratory tests with complete blood count, coagulation panel, comprehensive metabolic panel, liver function tests, renal function tests, and acute phase reactants revealed hemoglobin level of 8 grams/deciliter (normal level for males, 13.5 to 17.5 grams/decilitre), C-Reactive Protein (CRP) of 47.9 milligrams/liter (normal level less than 10 milligrams/liter), procalcitonin of 0.14 nanograms/milliliter (normal level less than 0.1 nanograms/milliliter), sodium of 127 milliequivalents/liter (between 135 and 145 milliequivalents/liter), lactate of 0.8 millimoles/liter (Normal lactate range is less than 2.3 millimoles/liter), the rest of the complete blood count, coagulation panel, comprehensive metabolic panel, liver function tests, and renal function tests were normal ( Figure 2). Arterial blood gases showed a partial pressure of oxygen of 57 mmHg and partial pressure of carbon dioxide of 27 mmHg.

**Figure 3.**
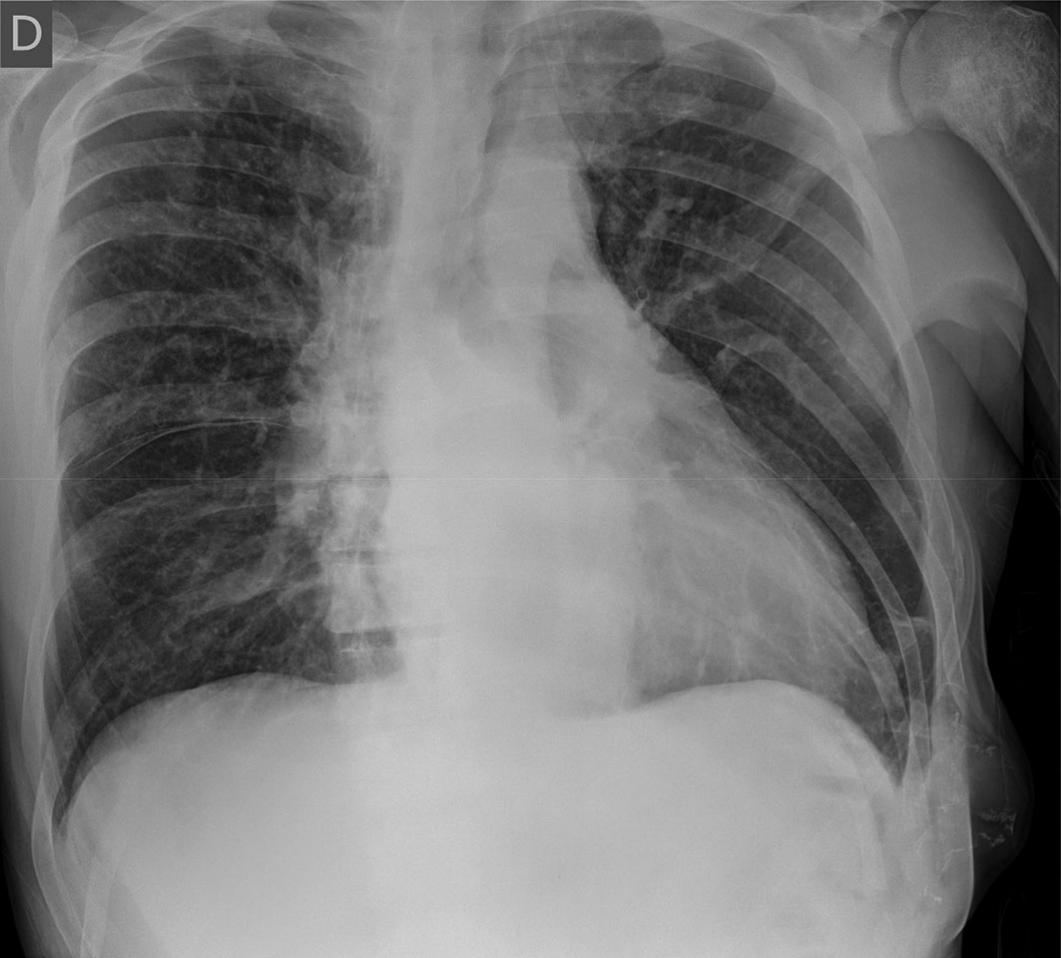
Chest radiograph at hospital admission.

**Figure 4.**
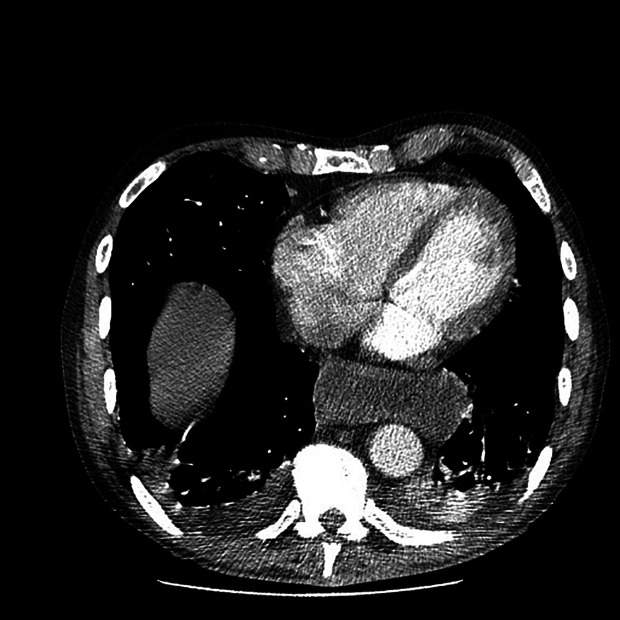
Computed tomography angiogram of the chest.

Cardiac enzymes and N-terminal pro-brain natriuretic peptide (NT-proBNP) were negative. An electrocardiogram showed no remarkable alterations. A chest radiograph ( Figure 3) exhibited an enlarged cardiac silhouette. A computed tomography angiogram of the chest ( Figure 4) was performed to confirm or rule out the possibility of a pulmonary embolism. It showed no signs of pulmonary embolism but confirmed an enlarged cardiac silhouette and exhibited a mild bilateral pleural effusion. Severe acute respiratory syndrome coronavirus 2 (SARS-CoV-2) polymerase chain reaction (PCR), influenza PCR, and *Streptococcus pneumoniae*, and *Legionella pneumophila* urinary antigen tests all came back negative. Finally, blood, urine, and sputum samples were collected for culture.

Based on the respiratory and heart rate, hypotension, CRP elevation, and fever, a serious infection could not be ruled out. Having a negative NT-proBNP did not point to a congestive failure despite imaging techniques. ^12^^,^^13^ Wide spectrum antibiotics with intravenous meropenem (2 grams/8 hours), oral linezolid (600 milligrams/12 hours), and intravenous trimethoprim/sulfamethoxazole (320/1600 milligrams/6 hours) as well as intravenous fluid therapy, and symptomatic treatment were started following hospital protocols. Oral dexamethasone previously prescribed for bone pain was increased to 4 mg twice a day as part of the treatment of acute respiratory insufficiency. The patient was transferred to the medical oncology inpatient ward.

During his stay, an echocardiogram was performed revealing a normal size left ventricle, with no segmentary alterations and a left ventricular ejection fraction within normal values.

Blood, urine, and sputum cultures were negative for bacterial and fungal pathogens. The medical team then requested a respiratory viruses PCR in nasopharyngeal exudate that displayed positivity for HBoV. The other viruses tested for (Influenza A, Influenza B, Influenza C, Parainfluenza 1, Parainfluenza 2, Parainfluenza 3, Parainfluenza 4, Enterovirus B, Rhinovirus, Coronavirus 229, Adenovirus, Metapneumovirus A, Metapneumovirus B, Respiratory syncytial virus A, Respiratory syncytial virus B) were negative. Based on the common concurrent infection rates with other pathogens, bronchoscopy was performed and bronchoalveolar lavage (BAL) was done to collect samples. Culture and a respiratory virus PCR were performed in BAL. The culture was negative for bacteria and fungi. The PCR was again, positive for HBoV. Antibiotic therapy was discontinued based on these results and progressive clinical and analytical improvement was shown with a gradual decrease in acute phase reactants.

**Figure 5.**
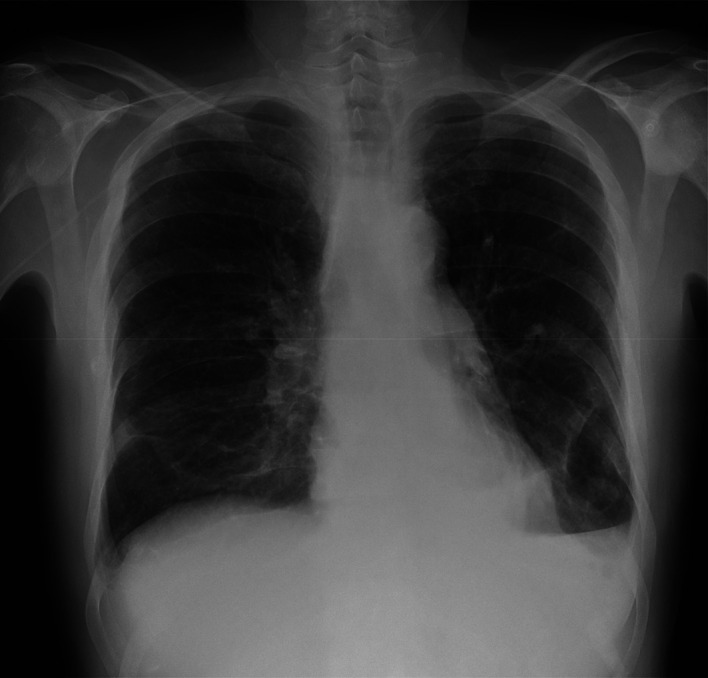
Chest radiograph at hospital discharge.

The patient gradually recovered from the dyspnea, chest pain and dry cough having no more episodes of fever during hospitalization. A second chest radiograph ( Figure 5) before discharge shows a normal size cardiac silhouette while maintaining the mild bilateral pleural effusion. After eight days from hospital admission, the patient was discharged home. He was re-evaluated two weeks after discharge in medical oncology outpatient clinic reporting no further complications with normal laboratory tests with complete blood count, comprehensive metabolic panel, and acute phase reactants.

## Patient perspective

I usually never complain but this time the pain and lack of breath were unbearable I could not keep waiting for these symptoms to go away. The first days I spent at the hospital I had lots of diagnostics tests done before the medical team could reach a diagnosis but at least they were done fast. During the time at the hospital started to feel better slowly but in the end, I could completely recover.

## Discussion

We report an infection with an emerging pathogen in a solid tumor patient. The case is rare as it presented contradictory diagnostic test results with an uncommon radiological image.

Emerging viral pathogens represent a growing threat for all people but especially for immunocompromised populations. Ultimately, we have seen an extremely prevalent example of this problem with the SARS-CoV-2. The coronavirus disease 2019 (COVID-19) pandemic takes a bigger mortality and morbidity toll when affecting cancer patients. ^14^

HBoV was first described in 2005, ^2^ however we do not have much information about its prevalence in patients suffering from solid neoplasms. With the one exception being the study from Li *et al* ^10^ that shows an almost 40% (62/156) HBoV prevalence among solid tumor patients compared to 3.51% (33/941) in children with respiratory tract infections. However, this study only reflects the population from Wuhan, and it does not have any clinical information regarding the patients enrolled. Meaning this data is likely to be different globally.

In a study by Lee *et al* ^11^ 185 patients infected with HBoV were enrolled. Of the 185 patients, 76 (41.08%) were immunocompromised. From the immunocompromised patients 28 (36.84%) suffered solid malignancies treated with chemotherapy within 6 months of HBoV diagnosis, 11 (14.47%) had hematologic malignancies, 19 (25%) had solid organ transplantations, and 18 (23.68%) received hematopoietic cell transplantation. Of the 185, 110 had pneumonia. CT findings were analyzed in 34 of the 185 patients, from which 16 were immunocompromised. There was no significant difference in CT patterns between immunocompetent and immunocompromised patients and the most frequent findings in both groups were bilateral consolidation and/or ground-glass opacities. Pleural effusion was observed in 50.0% of patients.

In our case report the patient presented with laboratory tests that pointed to an acute infectious process with a potential respiratory origin, however, imaging techniques showed conflicting data revealing an enlargement of the cardiac silhouette indicating a possible congestive heart failure. Vital signs, physical examination, a normal echocardiogram, clinical judgment, and the clinical picture all together tipped the balance towards an infectious diagnosis.

After the negative results in the sputum, urine, and blood culture, a PCR in nasopharyngeal exudate was performed, presenting a positive result for HBoV. Thus, revealing the probable cause of the patient's current pathologic process. Based on the fact that it is very common to have coinfections with other viruses HBoV had to be confirmed in BAL which revealed no other pathogen. ^5^ This finding was concordant with the bilateral pleural effusion based on available published articles ^11^ but not with the cardiac silhouette enlargement. After clinical improvement was noted a new chest radiography was performed that did not display an enlarged cardiac silhouette.

To our knowledge this is the first case report article of a solid tumor patient infected with HBoV. The discordant imaging and laboratory tests show the need for a clinical judgement that considers all aspects of the clinical picture and reinforces the necessity of further diagnostic efforts when diagnostic test results are conflicting. This is especially true in immunocompromised populations at risk of serious complications in case of a delayed diagnosis and treatment.

## Data availability

All data associated with this article are available in the paper and no additional source data is required.

## Consent

Written informed consent for publication of their clinical details, clinical images, and views on the treatment was obtained from the patient.
