# Supplementary material for: Case Report: Bocavirus Infection Radiologically Resembling a Congestive Heart Failure in a Patient with Metastatic Castration-Resistant Prostate Cancer
Source: F1000Res. 2022 Mar 29;11:196. Originally published 2022 Feb 16. [Version 2] doi: 10.12688/f1000research.109221.2 (PMC9021672; doi:10.12688/f1000research.109221.2)
Supplement: Peer review history

# Peer review: Reviewer response for version 2

**DOI:** 10.5256/f1000research.122669.r129132

**Published:** 2022-04-20

**Authors:** Marcos Roberto Tovani Palone

**Affiliations:** 1 Ribeirão Preto Medical School, University of São Paulo, Ribeirão Preto, Brazil

Authors should refine the use of the English language.

Are enough details provided of any physical examination and diagnostic tests, treatment given and outcomes?

Yes

Is the case presented with sufficient detail to be useful for other practitioners?

Yes

Is sufficient discussion included of the importance of the findings and their relevance to future understanding of disease processes, diagnosis or treatment?

Yes

Is the background of the case’s history and progression described in sufficient detail?

Yes

Reviewer Expertise:

Pathology, General medicine, Dentistry, Global and Public health

I confirm that I have read this submission and believe that I have an appropriate level of expertise to confirm that it is of an acceptable scientific standard.

# Peer review: Reviewer response for version 2

**DOI:** 10.5256/f1000research.122669.r129133

**Published:** 2022-03-29

**Authors:** Macarena Torrego-Ellacuría

**Affiliations:** 1 Department of Endocrinology and Nutrition, Hospital Clínico San Carlos, IdISSC, Madrid, Spain

The current version of the case report is adequate, I have no further comments to make

Are enough details provided of any physical examination and diagnostic tests, treatment given and outcomes?

Yes

Is the case presented with sufficient detail to be useful for other practitioners?

Yes

Is sufficient discussion included of the importance of the findings and their relevance to future understanding of disease processes, diagnosis or treatment?

Yes

Is the background of the case’s history and progression described in sufficient detail?

Yes

Reviewer Expertise:

Pharmacy, clinical research

I confirm that I have read this submission and believe that I have an appropriate level of expertise to confirm that it is of an acceptable scientific standard.

# Peer review: Reviewer response for version 1

**DOI:** 10.5256/f1000research.120698.r123948

**Published:** 2022-03-17

**Authors:** Marcos Roberto Tovani Palone

**Affiliations:** 1 Ribeirão Preto Medical School, University of São Paulo, Ribeirão Preto, Brazil

This is an interesting article that describes and discusses the case of a 59-year-old patient with a history of metastatic castration-resistant prostate cancer suffering from HBoV infection.

 The article is well structured, with a complete description of the case and relevant discussion. Furthermore, the references used are up to date and appropriate and the figures are very illustrative.

 However, revisions are required to refine the article. Authors should reread the article in an attempt to review it for grammatical mistakes and writing style.

Are enough details provided of any physical examination and diagnostic tests, treatment given and outcomes?

Yes

Is the case presented with sufficient detail to be useful for other practitioners?

Yes

Is sufficient discussion included of the importance of the findings and their relevance to future understanding of disease processes, diagnosis or treatment?

Yes

Is the background of the case’s history and progression described in sufficient detail?

Yes

Reviewer Expertise:

Pathology, General medicine, Dentistry, Global and Public health

I confirm that I have read this submission and believe that I have an appropriate level of expertise to confirm that it is of an acceptable scientific standard, however I have significant reservations, as outlined above.

# Response

**Published:** 2022-03-20

**Authors:** Javier David Benitez Fuentes

**Affiliations:** San Carlos University Hospital, Spain

Article has been reread by various author correcting minor mistakes and improving writing style. We have also added one image from the computed tomography angiogram of the chest to increase the visual aspect of the case.

# Peer review: Reviewer response for version 1

**DOI:** 10.5256/f1000research.120698.r125565

**Published:** 2022-03-09

**Authors:** Macarena Torrego-Ellacuría

**Affiliations:** 1 Department of Endocrinology and Nutrition, Hospital Clínico San Carlos, IdISSC, Madrid, Spain

Interesting, detailed, and justified article. Some minor suggestions to improve:

1. Introduction, third paragraph, line 1: change " *articles published*" for " *published articles*" (structure adjective + noun). 
2. Introduction, third paragraph, penultimate line. Replace " *From these 185*" with " *Of these 185, 28 (15,13%) had solid tumours*". 
3. Use the International System of Units (SI) symbols for the expression of all dose or concentrations related to biological samples, always leaving a space between the figure and the symbol used (pages 3,4,5, and figure 2). For the pharmacological treatment regimen (e.g. 2 grams every 8 hours or 600 milligrams every 8 hours) the current expression can be retained, without abbreviations. 
4. Figure 1: include figure legend with abbreviations used in the figure. 

Are enough details provided of any physical examination and diagnostic tests, treatment given and outcomes?

Yes

Is the case presented with sufficient detail to be useful for other practitioners?

Yes

Is sufficient discussion included of the importance of the findings and their relevance to future understanding of disease processes, diagnosis or treatment?

Yes

Is the background of the case’s history and progression described in sufficient detail?

Yes

Reviewer Expertise:

Pharmacy, clinical research

I confirm that I have read this submission and believe that I have an appropriate level of expertise to confirm that it is of an acceptable scientific standard.

# Response

**Published:** 2022-03-19

**Authors:** Javier David Benitez Fuentes

**Affiliations:** San Carlos University Hospital, Spain

From your suggestions, I have followed points 1, 2, 4. Point 3 was done this way based on the comments from the editors of the journal so I will keep them that way. Thank you
